# Exercise-induced extracellular vesicles in reprogramming energy metabolism in cancer

**DOI:** 10.3389/fonc.2024.1480074

**Published:** 2025-01-06

**Authors:** Marju Puurand, Alicia Llorente, Aija Linē, Tuuli Kaambre

**Affiliations:** ^1^ Laboratory of Chemical Biology, National Institute of Chemical Physics and Biophysics, Tallinn, Estonia; ^2^ Department of Molecular Cell Biology, Institute for Cancer Research, Oslo University Hospital, Oslo, Norway; ^3^ Centre for Cancer Cell Reprogramming, Faculty of Medicine, University of Oslo, Oslo, Norway; ^4^ Department for Mechanical, Electronics and Chemical Engineering, Oslo Metropolitan University, Oslo, Norway; ^5^ Cancer Biomarker group, Latvian Biomedical Research and Study Centre, Riga, Latvia

**Keywords:** extracellular vesicles, cancer, energy metabolism, cancer microenvironment, physical exercise

## Abstract

Cancer is caused by complex interactions between genetic, environmental, and lifestyle factors, making prevention strategies, including exercise, a promising avenue for intervention. Physical activity is associated with reduced cancer incidence and progression and systemic anti-cancer effects, including improved tumor suppression and prolonged survival in preclinical models. Exercise impacts the body’s nutrient balance and stimulates the release of several exercise-induced factors into circulation. The mechanisms of how exercise modulates cancer energy metabolism and the tumor microenvironment through systemic effects mediated, in part, by extracellular vesicles (EVs) are still unknown. By transferring bioactive cargo such as miRNAs, proteins and metabolites, exercise-induced EVs may influence cancer cells by altering glycolysis and oxidative phosphorylation, potentially shifting metabolic plasticity – a hallmark of cancer. This short review explores the roles of EVs in cancer as mediators to reprogram cellular energy metabolism through exchanging information inside the tumor microenvironment, influencing immune cells, fibroblast and distant cells. Considering this knowledge, further functional studies into exercise-induced EVs and cellular energy production pathways could inform more specific exercise interventions to enhance cancer therapy and improve patient outcomes.

## 1 Introduction

Most cancers arise from a complex etiology involving genetic, environmental and lifestyle factors and their interactions (1, 2), and there is a great need and opportunity for cancer prevention through lifestyle change. Numerous epidemiological studies have provided evidence supporting the link between higher physical activity levels and a lower incidence of multiple cancer types (3, 4). Systemic reviews of the preclinical animal studies have demonstrated the anti-cancer effect (a decrease in cancer incidence and tumor size and a prolonged life span of animals) of physical exercise (5–7). The exact doses, types and routines/protocols of physical activity in decreasing cancer risk and mortality continue to be under active investigation. Elucidation of the mechanisms linking bodily activity interventions to cancer may have great potential in fighting this debilitating disease.

Profound changes in both cell genome and gene expression during malignant transformation are likely to be accompanied by changes in metabolism, including pathways for cellular energy production (8). The metabolomic analysis of human patients’ colorectal cancer xenografts in mice models revealed exercise-induced changes in intratumoral central carbon metabolism (especially mitochondrial metabolism), independently of whether exercise inhibited tumor growth (9). Investigating the mechanisms of exercise`s effect on cancer cells and tumors is complicated. This is because cancer cells inside the tumor are heterogeneous, and in addition they are surrounded and dependent on the functioning of cells forming the tumor microenvironment (10, 11), which can also be affected by exercise.

In general, the explanations of how exercise and muscle-derived bioactive molecules secreted into circulation can affect cancer cells (summarizing the review by Kang et al. (12)) are (1) enhancement of cancer immunosurveillance through increased innate immune function to boost the body’s ability to detect tumor cells and inhibit their growth; (2) regulation of inflammation and oxidative stress in conjunction with immune functions; (3) activation of physiological mechanisms such as changes in nutrient availability and metabolism. In parallel, physical exercise can stimulate tumor vascularization and improve the infiltration of systemic biomolecules and immune cells, thus affecting the entire tumor microenvironment.

One of the communication forms between cells across the body is via extracellular vesicles (EVs). EVs are lipid bilayer-enclosed nano to micro-sized particles that cannot replicate independently and are released by virtually all cell types in the body (13). Mounting evidence supports the hypothesis that EVs are not a random waste elimination mechanism but mediate intercellular communication, allowing the specific intercellular exchange of proteins, lipids, and genetic material (14, 15). Several recent studies have shown that the amount of circulating EVs rapidly increases due to physical exercise (16–18). According to the study by Frühlbeis et al., 2015, the increase in plasma EV peaks at 10 min after acute exercise, starts to decline within 30 min after training and returns to the baseline levels after 90 min (16). However, the increase in EV levels after exercise does not seem to be related to exercise intensity per se (16, 19). It has been suggested that EVs released from contracting skeletal muscles play a central role in mediating the systemic benefits of exercise by transporting bioactive molecules, which account for only about 5% of plasma EVs, although EVs released by immune cells contribute more (20). The results of several studies with human subjects suggest that exercise affects the content of circulating EVs in the blood (16–18, 21–23). A comprehensive review of the content of exercise-induced EVs (especially regarding nucleic acid content) can be found in Llorente et al., 2024 (23). Exercise-conditioned serum and myokines have been suggested to impact several hallmark features of cancer and act as tumor suppressors in breast, colon and lung cancer (24, 25). The role of EVs released into the systemic circulation during exercise in influencing the reprogramming of cancer energy metabolism is unknown. Considering the current understanding of EV circulation kinetics, the systemic effects of exercise-induced EVs seem to be relatively short-lived. However, localized increases in EVs within tissues and tumors may have more enduring effects and influence cellular energy metabolism.

The EVs secreted by tumors are abundant in plasma, and their use to find biomarkers for assessing disease progression or treatment efficacy is widely explored (26, 27). It also has to be considered that during exercise, the same stimuli that trigger the EV release from normal cells could also stimulate the release of EVs from cancer cells. However, this can have unintended consequences. EVs released by drug-resistant tumor cells or neighboring cells from the tumor microenvironment have an established role in the intercellular transfer of drug-resistance traits, with accompanying metabolic reprogramming, to drug-sensitive tumor cells (28) as reviewed by Polonia and Xavier (29, 30). In this context, a detailed knowledge of how different exercise types and doses affect the energy metabolism of cancer cells and other cells in the cancer microenvironment could help determine the type of exercise suitable for the patient during treatment, allowing better use or mimicking of the positive effects of exercise. Investigating the role of EV cargo in the modulation of tumor energy metabolism represents a new opportunity to target and stop disease development, particularly regarding antibodies’ against specific putative membrane receptors.

This mini-review analyzes examples of functional studies of how EV content can reprogram recipient cell glycolysis and OXPHOS in cancer. This knowledge also reflects on the potential role of exercise-induced EVs in reprogramming cellular energy metabolism in cancer.

## 2 Reprogramming cellular energy metabolism in cancer: metabolic plasticity and intercellular cooperation in the tumor microenvironment

Changes in cell metabolism contribute to transformation and tumor progression. There are only two central energy-producing metabolic pathways, glycolysis and oxidative phosphorylation (OXPHOS), which are, at the same time, sources for essential precursors for nucleotide, polysaccharides, amino saccharides, amino acids and lipid biosynthesis (31–33). The constant need of cancer cells for resources to ensure proliferation keeps both pathways working in cancer cells as well, but often, the proportion of usage and partly also the direction of the pathway is different in cancer cells than in normal tissue cells (34).

The primary bioenergetic profile of a cancer cell, which is different from its progenitor, may be as follows: (1) enhanced glycolysis under any conditions (Warburg effect); (2) unlike normal cells, cancer cells utilize both anaerobic and aerobic energy production with increased capacity (a hybrid phenotype); (3) be mainly OXPHOS users (34, 35). In addition, unlike non-neoplastic cells, a striking feature of cancer cells is their metabolic plasticity – the ability to readily switch their metabolic phenotypes to glycolysis or OXPHOS in response to changes in the microenvironment (*i.e.*, variations in O_2_ concentration, lactate concentration, pH, carbon source and growth factors availability) or inhibition of one of these pathways, giving survival advantages during tumor progression (34, 36). This metabolic plasticity is promoted by the hybrid bioenergetic phenotype and is linked with metastasis and chemoresistance (35). However, it is still not fully understood how cancer cells regulate gene expression to maintain their hybrid energy metabolism state, although variations in the expression of key transcriptional factors and oncogenes like HIF-1α, p53, c-Myc, PGC-1α are clearly involved (37). Elucidation of intrinsic and extrinsic factors (e.g., EVs) in the metabolic reprogramming of cancer cells is beneficial for better treatment of disease.

There is a growing recognition that the metabolism of cells other than cancer cells within the tumor microenvironment, like endothelial cells, fibroblasts and immune cells, can modulate tumor progression, including supporting and shaping the metabolism of cancer cells (38, 39). In addition to the soluble factors found in the tumor microenvironment, cancer cells and their energy metabolism are also targets for active communication with EVs released into the tumor microenvironment by surrounding cells, mainly by cancer-associated fibroblasts (CAFs) and tumor-associated macrophages (TAMs) (40, 41). Indeed, in breast tumors, cancer cells and CAFs may establish the coupled metabolic pattern sometimes referred to as the „reverse Warburg effect”, by which glycolytic CAFs provide energy-rich metabolites like lactate and glutamine to fuel the mitochondrial respiration and anabolic metabolism of cancer cells (42). Mechanisms of this setup also involve EVs secreted into the tumor microenvironment by all cells involved.

There is evidence that patient-derived CAFs can extensively reprogram the energy metabolism of the recipient cancer cell in culture with EVs only (43). The relatively high abundance of miRNAs that target OXPHOS genes resulted in decreased mitochondrial respiration, upregulated glycolysis and enhanced reductive pathway of glutamine metabolism (reductive carboxylation) in prostate cancer cells cultivated with EVs released from prostate cancer patient-derived CAFs (43). Supplementation of cellular nutrition through the secretion of metabolites is another possible mechanism besides miRNAs in how the EVs from CAFs affect the metabolism of recipient tumor cells. Exosomes released by CAFs derived from prostate cancer patients are a vital source of metabolites that are essential contributors to central carbon metabolism under conditions of nutrient deprivation (43).

In turn, EV-packed miRNAs derived from cancer cells could be critical agents modulating the energy metabolism of target cells in the tumor microenvironment. For example, the breast cancer cell-secreted and EV-encapsulated miR-105 reprograms energy metabolism in CAFs (44). According to the model, a high MYC activity in cancer cells leads to a high secretion of miR-105 to activate MYC signaling in stromal cells, phenotypically extending the effects of MYC originating from cancer cells to non-cancer cells. The miR-105-EV-reprogrammed stromal cells increased the catabolism of glucose and glutamine to fuel adjacent cancer cells when nutrients were sufficient. When nutrients are in shortage and metabolic by-products accumulate, these reprogrammed CAFs detoxify metabolic wastes, including lactic acid and ammonium, by converting them into fuel metabolites, making them substrates for the surrounding cancer cells (44).

It has been suggested that one of the possible mechanisms of exercise for cancer prevention or in inhibiting cancer development might be the intervention of the regulation of TAM polarization (45). TAM polarization is a process actively driven by cancer cell-derived EVs. A previous investigation revealed that EVs derived from human hepatocellular carcinoma carrying glycolytic enzyme pyruvate kinase M2 isoform (PKM2) foster the progression of carcinoma by promoting PKM2 polarization of macrophages and reconfiguring the tumor immune microenvironment (46). A similar phenomenon is demonstrated also in gastric cancer (47). Wu et al. showed that gastric cancer cells can deliver PKM2 packed into EVs to macrophages, leading to the differentiation of macrophages into the pro-inflammatory M2 subtype, consequently promoting the progression of gastric cancer. In addition, in a study involving patients with neuroblastoma, glycolytic enzymes PKM2 and Hexokinase II were detected in circulating EVs isolated from blood using mass spectrometry (48). Overall, tumor cell-derived PKM2 could promote macrophage differentiation through glycolytic reprogramming. However, the non-metabolic functions of PKM2, such as its activity as a protein kinase or transcriptional coactivator (49, 50), could also participate in metabolic reprogramming. How PKM2 levels in EVs are affected by exercise needs further investigation.

An example is also found in EVs from TAMs’ participation in reprogramming breast cancer cell metabolism (51). Primary TAMs isolated from breast cancer patients were co-cultured with breast cancer cells without physical contact between them. TAM-derived EVs enhance breast cancer cells’ glycolysis and apoptotic resistance by transmitting long noncoding RNAs that stabilize HIF1-alpha and enhance glycolysis in the target cells (51). Unfortunately, the function of OXPHOS was not evaluated in this study. However, there is a lack of studies showing the direct functional effect of factors contained in EVs on the energy metabolism of recipient cancer cells. The profile of EVs probably also affects the metabolic plasticity of cancer cells, which changes more unfavorable for cancer. Functional investigation of mechanistic basis and intercellular codependencies in tumor microenvironments may allow the identification of specific vulnerabilities and targets for eradicating cancer cells. How physical exercise affects these relationships is a challenging research topic for the future.

## 3 Effects of physical exercise-induced EVs on cancer energy metabolism

Physical activity (particularly intense exercise) affects the whole body, is safe for patients, and improves their quality of life. Exercise-induced overall systemic effects on cellular bioenergetics can be realized through fluctuations in nutrient balance. At the molecular level, an exercise-induced deficit of nutrients such as glucose and glutamine downregulates intrinsic cellular cancer-promoting signaling pathways like PI3K/AKT/mTOR and RAS-ERK and thereby increases AMP-activated protein kinase (AMPK) activity (52, 53). As AMPK acts as a master regulator of OXPHOS and mitochondrial biogenesis (54), which generally inhibits anabolism under energetic stress, the bioenergetic consequences of such changes involve the downregulation of (aerobic) glycolysis and activation of mitochondrial biogenesis and OXPHOS (55).

**Figure 1:**
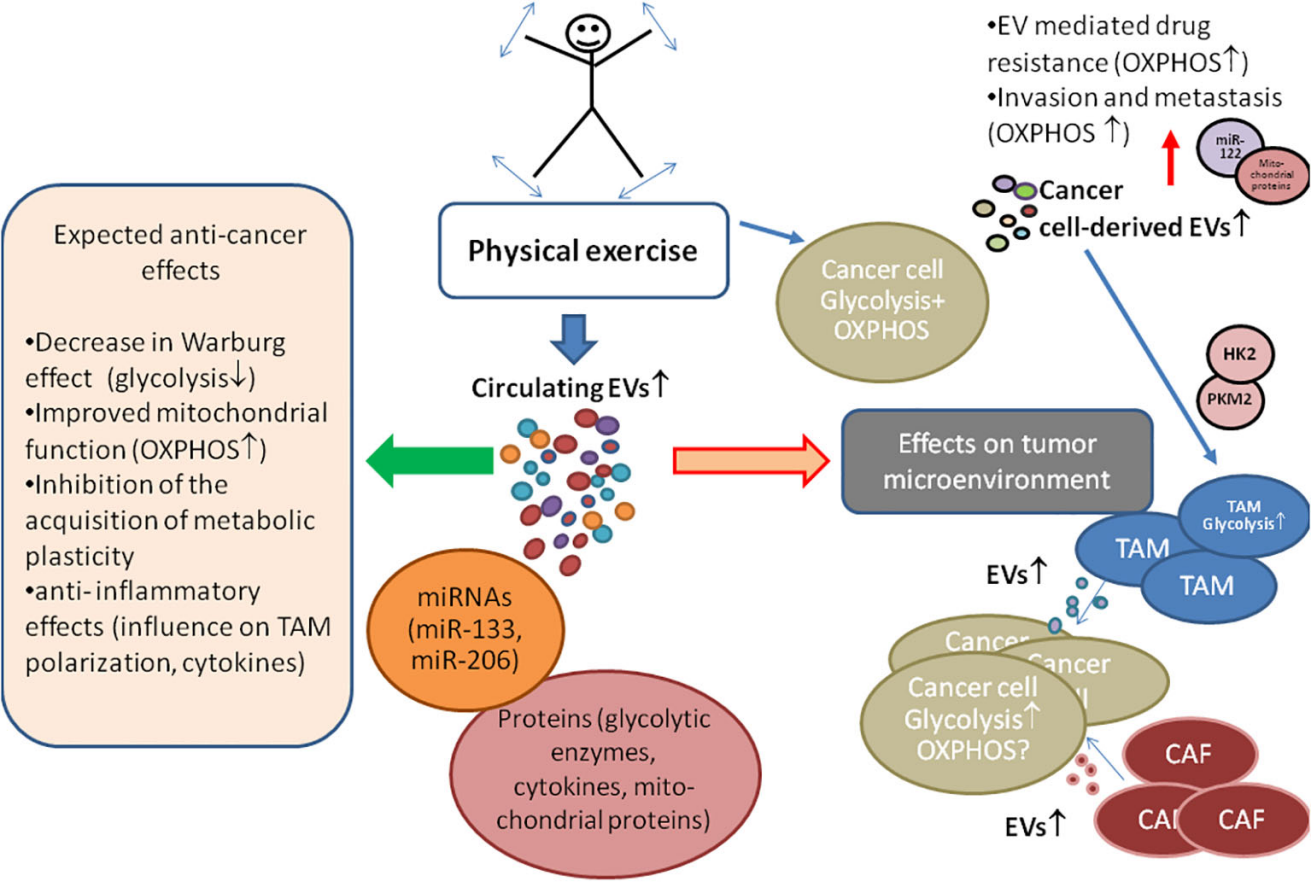
The role of exercise-induced extracellular vesicles (EVs) in cancer energy metabolism and tumor microenvironment. Experimental animal studies have consistently reported that physical exercise leads to a reduction in cancer incidence, a decrease in tumor size, and a prolonged life span of animals, suggesting similar benefits for humans. Exercise prompts various cells, including cancer cells, to release EVs containing bioactive molecules like glycolytic enzymes (pyruvate kinase II (PKM2), hexokinase II (HK2)), mitochondrial proteins, and miRNAs, which influence recipient cell metabolism. Cancer energy metabolism is often characterized by high glycolysis activity, with oxidative phosphorylation (OXPHOS) also not being defective. Unlike normal cells, cancer cells acquire the ability to switch their primary energy production pathway, i.e., they acquire metabolic plasticity supporting growth and drug resistance. EVs secreted by tumor microenvironment cells, such as cancer-associated fibroblasts (CAF) and tumor-associated fibroblasts (TAM), can enhance glycolysis in cancer cells. However, cancer cell-derived EVs may have a pro-OXPHOs effect (demonstrated through miR-122 and mitochondrial component exchange), contributing to drug resistance and supporting cellular metabolic plasticity, effects that can be minimized with the proper exercise regimens. The symbols ↓,↑ indicate up- and down-regulation, respectively.

Other mechanisms influencing metabolism are driven by agents secreted by cells during exercise. The results of several studies with human subjects suggest that exercise affects the content of circulating EVs in the blood (16–18, 21, 22). Further, exercise-induced EVs also carry active enzymes, critical regulatory factors and metabolites that affect OXPHOS and glycolysis fluxes (**Figure 1**), researching the effects of which would help implement exercise programs that support cancer treatment.

A recent study demonstrated that exercise induces the secretion of over 300 proteins into EVs, including glycolytic enzymes and cytokines secreted by skeletal muscle (18). Proteome data show that small EV-delivered glycolytic enzymes can influence the glycolysis rate of recipient cells (56), but the exact extent of such changes is not well known.

Within the various exercise- and cancer-related EV cargos, miRNAs are some of the most extensively studied candidates for mediating specific muscle-to-cancer crosstalk (57–59). Among the exercise-induced miRNA families, miR-1, -133 and -206 are the most widely investigated (60, 61). In several studies, members of these families have been shown to be enriched in EVs released after exercise (21, 62–64). However, miR-1, miR-133a, miR-133b and miR-206 are primarily down-regulated in various cancers where they function as tumor suppressors (65, 66). A direct effect on cancer energy metabolism has only been shown for miR-133b. The regulatory network of miR-133b is involved in the altered energy metabolism of cancer cells. MiR-133b controls pyruvate kinase expression, thereby repressing glycolysis (67–69), a property that can confuse the metabolism of cancer cells. This process reduces the supply of pyruvate, which may decrease OXPHOS activity along with higher production of oxygen free radicals. Under these conditions, cancer cells could become vulnerable to treatments that induce oxidative stress, such as doxorubicin or radiation.

So far, glycolysis in cancer has received more attention. However, OXPHOS is also active in cancer cells, which has yet to be directly measured and investigated. Different approaches involving high-resolution respirometry in cells and tissues allow the determination of OXPHOS activity and efficiency and cancer-specific alterations in OXPHOS donor pathways such as the Krebs cycle (70, 71). Such studies in the context of exercise-induced EVs would contribute to implementing the positive effects of exercise in clinical practice.

## 4 Mitochondrial components in EVs

Mitochondria are dynamic organelles, and the constant remodeling of mitochondria is driven mainly by the outcome of continuous maintenance and quality control. The number of mitochondria and their activity can change in response to various physiological or pathological conditions. As a part of routine mitochondrial maintenance, damaged mitochondria undergo fission, and resulting vesicles are directed to mitophagy by lysosomes (72). In various cell types, the generation of mitochondria-derived vesicles is accelerated by oxidative stress and their cargo is enriched with oxidized proteins (73, 74). As the biogenesis of mitochondria-derived vesicles is faster than fission, resulting vesicles are likely to be the first round of defense for the mitochondria to eject damaged proteins and avoid the complete failure of the organelle (75, 76). Therefore, it is unsurprising that specific EV subpopulations isolated from blood plasma have been found to contain mitochondrial proteins, fragments, and, in some cases, full-length mitochondrial DNA (77).

Consequently, an increase in mitochondrial proteins in EVs can reflect the presence and degree of disease. Notably, some evidence suggests that EVs isolated from the blood plasma of cancer patients are enriched with mitochondrial membrane proteins compared to EVs derived from control patients (15). Plasma EVs from breast cancer patients contain higher levels of mitochondrially encoded mRNAs for subunits of NADH dehydrogenase, ATP synthase, and cytochrome C oxidase (78). Enrichment of plasma EVs with mitochondrial proteins and mRNAs in cancer suggests that mitochondria-derived vesicles may play an important role in modulating the cellular stress response.

However, which cells exactly produce these vesicles and whether these vesicles also have specific recipients with specific receptors is not known.

At the local level, within the tumor microenvironment, there is evidence of the intercellular transfer of mitochondria as an additional mechanism to replace damaged mitochondria. Mitochondrial transfer between astrocytes and neurons has been observed after acute stress like an ischemic insult (79), from mesenchymal stem cells to injured pulmonary epithelial cells (80–82), both *in vivo* and *in vitro*, and from bone marrow stromal cells to acute myeloid leukemia cells during chemotherapy (83). Mitochondrial transfer between mesenchymal stem cells, endothelial cells, and cancer cells by tunneling via nanotubes occurs probably more often than one might think and is an influencing factor in cancer treatment by promoting the development of chemoresistance (84–88).

The cancer-promoting effect of mitochondria transfer is suggested to occur mainly by potentiating OXPHOS through the influx of mutation-free mitochondrial DNA, which ensures efficient energy production and reduces oxygen free radical generation (82, 89). How exercise-induced EVs might interfere with intercellular mitochondrial information transfer remains unknown.

## 5 EVs and cancer-to-muscle crosstalk in developing cancer cachexia

Rapid and progressive loss of muscle mass (sarcopenia) is a distinctive characteristic of a cancer-related multifactorial syndrome called cachexia (90). Decreased mitochondrial OXPHOS and mitochondrial damage are linked to diminished muscle function and muscle weakness in cancer patients (91–93). Altered mitochondrial morphology, decreased interaction of mitochondria with endoplasmic reticulum, decreased ATP synthesis, and augmented uncoupling seem to be the main trends associated with skeletal muscle mitochondria during cancer states (91, 93, 94). Poor muscle quality and low muscle mass have been related to worse treatment outcomes and higher mortality in patients with metastatic and nonmetastatic breast cancer (95–97). Sarcopenia is reported in one-third (34%) of nonmetastatic, newly diagnosed breast cancer patients before chemotherapy or radiation (95). Also, the extent of the association of low muscle mass with poor survival is similar in stage II and III breast cancer (95). In addition, there is no relationship between muscle gene expression and anti-cancer treatment (92).

Evidence shows that changes in metabolism and aspects of the inflammatory response in cancer-associated muscle wasting is modulated by miRNA-containing EVs (58, 98, 99). It has been shown that breast cancer cell-derived EVs containing miR-122 play an important role in reprogramming systemic energy metabolism, including mitochondrial metabolism in skeletal muscle (100–102). The levels of miR-122 in total circulating EVs were higher in the breast cancer group compared to the control group (102). EVs containing miR-122 released by breast cancer MDA-MB-231 cells suppressed p53 signaling in skeletal muscle cells. Consequently, suppression of p53 signaling by miR-122 decreased the expression of TP53 target genes related to mitochondrial homeostasis, like Tfam, Pgc-1a, Sco2, and 16S ribosomal RNA, resulting in impaired energy production in skeletal muscles, promoting muscle deconditioning and cancer-associated cachexia development (101).

The same breast cancer cell-derived miR-122 can modify potential pre-metastatic niches in the lung and brain by downregulating glucose uptake and glycolysis by decreasing pyruvate kinase and GLUT1 expression and increasing the local nutrient availability for metastatic cancer cells (100). However, another study shows that higher levels of circulating miR-122 were specifically associated with predicting metastatic recurrence in stage II-III breast cancer patients (103). The effects of miR-122 may explain the production of specific miRNA-loaded EVs, which is the reason for the heterogeneity of metastasis and cachexia. How and to what extent a patient’s physical activity or different nutritional and conditional settings mimicking exercise-induced changes affect miRNA production in cancer cells requires further investigation.

## 6 Limitations and challenges for further research

Since EV content and type of generation are intrinsically linked to cellular processes, they offer potential as biomarkers or therapeutics for a range of diseases (104).

Limitations and challenges in the study of EVs are primarily technical. The lack of methods to rapidly and reliably quantify and trace EVs from different origins makes studying the effects of exercise-induced EVs challenging. Due to the exosome heterogeneity, non-standardized isolation methods and proteomics/bioinformatics approaches (77), the information about the EV-s in intercellular communication is still unclear. The underlying molecular mechanisms of exercise training in cancer remain poorly understood. The incomplete nomenclature of EVs also limits the understanding of their functional role in cellular physiology (14).

The main challenges in determining the general effects of exercise on subjects include individual differences, comorbidities, and the wide variety of exercise types and loads. Continued technological advancements, alongside more standardized approaches, will be essential for addressing these limitations and furthering our understanding of the molecular mechanisms underlying the effects of exercise and disease.

## 7 Conclusions

Physical exercise stimulates cells to release EVs, and without a clear understanding of the specific EVs induced by exercise in cancer patients and their effects, it is difficult to recommend exercise with complete confidence. That is because EVs released from cancer cells have several effects, which could complicate the course of the disease. Regarding exercise in the context of cancer energy metabolism, it remains unclear which EVs are beneficial allies and which act as harmful foes.

We are just beginning to explore direct associations between muscle health, exercise, and cancer cells and discovering complex cooperation mechanisms between different cell types in tumors. By studying the factors, including EVs, that affect the energy metabolism of cancer cells, we can target malignant cells with higher precision. The miRNAs, along with mitochondrial and glycolytic proteins present in EVs released into the tumor microenvironment and circulation, can influence the metabolic pathways utilized for energy production in recipient cells. Consequently, EVs released by cells after exercise certainly have the potential to affect the metabolism of the recipient cell. However, functional studies of cellular energy metabolism, which include measurements of OXPHOS and glycolysis fluxes of tumor tissues or cells, shall provide a better understanding of how to attack cancer cells and reverse the cancer-specific reprogramming of energy metabolism. This knowledge is essential for enhancing cancer treatment by amplifying or replicating the beneficial effects of exercise.
